# Ten simple rules for organizing a bioinformatics training course in low- and middle-income countries

**DOI:** 10.1371/journal.pcbi.1009218

**Published:** 2021-08-19

**Authors:** Benjamin Moore, Patricia Carvajal-López, Paballo Abel Chauke, Marco Cristancho, Victoria Dominguez Del Angel, Selene L. Fernandez-Valverde, Amel Ghouila, Piraveen Gopalasingam, Fatma Zahra Guerfali, Alice Matimba, Sarah L. Morgan, Guilherme Oliveira, Verena Ras, Alejandro Reyes, Javier De Las Rivas, Nicola Mulder

**Affiliations:** 1 European Molecular Biology Laboratory, European Bioinformatics Institute, Wellcome Genome Campus, Hinxton, Cambridge, United Kingdom; 2 Computational Biology Division, Department of Integrative Biomedical Sciences, Institute of Infectious Disease and Molecular Medicine, CIDRI Africa Wellcome Trust Centre, Faculty of Health Sciences, University of Cape Town, Cape Town, South Africa; 3 Vicerrectoria de Investigación y Creación, Universidad de los Andes, Bogotá, Colombia; 4 Institut Français de Bioinformatique, Centre National de la Recherche Scientifique, Paris, France; 5 Unidad de Genómica Avanzada (Langebio), Centro de Investigación y de Estudios Avanzados del IPN, Guanajuato, Mexico; 6 Institut Pasteur de Tunis, Laboratory of Transmission, Control and Immunobiology of Infections, Tunis-Belvédère, Tunisia; 7 Wellcome Connecting Science, Wellcome Genome Campus, Hinxton, Cambridge, United Kingdom; 8 Instituto Tecnológico Vale, Belém, Pará, Brazil; 9 Universidad de los Andes, Max Planck Tandem Group in Computational Biology, Department of Biological Sciences, Universidad de los Andes, Bogotá, Colombia; 10 Cancer Research Center, Consejo Superior de Investigaciones Científicas and University of Salamanca, Campus Miguel de Unamuno, Salamanca, Spain; Dassault Systemes BIOVIA, UNITED STATES

## Introduction

Bioinformatics training is required at every stage of a scientist’s research career. Continual bioinformatics training allows exposure to an ever-changing and growing repertoire of techniques and databases, and so biologists, computational scientists, and healthcare practitioners are all seeking learning opportunities in the use of computational resources and tools designed for data storage, retrieval, and analysis.

There are abundant opportunities for accessing bioinformatics training for scientists in high-income countries (HICs), with well-equipped facilities and participants and trainers requiring minimal travel and financial costs alongside a range of general advice for developing short bioinformatics training courses [1–3]. However, regionally targeted bioinformatics training in low- and middle-income countries (LMICs) often requires more extensive local and external support, organization, and travel. Due to the limited expertise in bioinformatics in LMICs in general, most bioinformatics training requires a fair amount of collaboration with experts beyond the local community, country, or region. A common model of training, used as the basis of this article, includes a local host collaborating with local, regional, and international experts gathering to train local or regional participants.

Recently, there has been a growth of capacity strengthening initiatives in LMICs, such as the Pan African Bioinformatics Network for Human Heredity and Health in Africa (H3ABioNet) Initiative [4–6], the Capacity Building for Bioinformatics in Latin America (CABANA) Project [7], the Asia Pacific BioInformatics Network (APBioNet) [8], and the Wellcome Connecting Science Courses and Conferences program [9]. One of the important strands of these initiatives is a drive to organize and deliver valuable bioinformatics training, but organizing and delivering short bioinformatics training workshops in an LMIC present a unique set of challenges. This paper attempts to build upon the sage advice for organizing bioinformatics workshops with specific guidance for organizing and delivering them in LMICs. It describes the processes to follow in organizing courses taking into consideration the low-resource setting.

We should also note that LMICs are not a monolithic group and that setting, context, temporality, and specific location matters. LMICs are a complex regional grouping [10] and should be treated as such; however, we will present some common lessons that we hope will help organizers and trainers of bioinformatics training events in LMICs to navigate the often different, challenging, and rewarding experience.

### Rule 1: Define the topic, aims, and the target audience of the training course

Developing a course that delivers an effective learning experience is dependent on your ability to meet the trainees’ needs while also providing the framework for long-term impact, sustainability, and community building. This is of particular importance in LMICs where there may not be an extensive existing training capacity or a critical mass of expertise to organize and deliver training. Therefore, the organizers must be engaged with the long-term aims of the training event within the local scientific community as well as the trainees’ needs and research interests. This knowledge will help you define SMART learning objectives [11], which can be based on existing competency frameworks, such as the framework developed by the International Society for Computational Biology (ISCB) [12] and Bloom’s Taxonomy [13].

Although it may seem natural that defining the topic, aims, and target audience should be the first step when planning a course or workshop, this is often an iterative process that requires time and organization. Initial assessment of the local trainees’ needs, including topics/subject area of interests, skill gaps, and expectations, is crucial. Further information such as availability of resources, data access, and capacity to deliver the training should be considered at this stage too. Such information can be obtained through expert consultation, surveys, and available literature. Clearly defining the trainees’ needs and available resources should guide the generation of specific questions in an application form to evaluate applicants’ knowledge level and expertise, which will, in turn, allow you to better identify gaps in knowledge and orient the generation of the training goals accordingly. Together with budget and time considerations (see Rule 2), this research will allow you to define the scope of the course to address the identified knowledge and skills gaps.

### Rule 2: Applying for funding requires a clearly defined workshop outline and budget

Delivering bioinformatics training requires a carefully considered budget. The cost of your training will be highly dependent on your training topics and format. Defining the scope and desired learning outcomes for your training at an early stage in the planning process (see Rule 1) will dictate the length and format of the training, and, therefore, provide an indication of the required budget. Reciprocally, budgetary constraints may dictate the scope, length, and format of the training. Thus, budget and workshop structure are intertwined and should inform each other throughout the planning stage.

While some training events simply require a training room and participants to be present (low cost), advanced training often requires dedicated IT infrastructure, experienced trainers, and support staff (higher cost). In the absence of a well-planned budget, training costs can quickly escalate, leading to a negative training experience for both trainers and participants. A carefully planned and considered budget drawn up well ahead of the training enables funding to be sourced and secured in good time, reducing the potential for this negative experience.

When formulating the budget, it is important to consider costs associated with the venue, catering, travel, and IT infrastructure. A detailed budget template can be found in the H3AbioNet training guide, along with other useful templates and guides [14]. The most costly items often include course venue, travel, and accommodation. Obtain general quotations from various providers for comparisons. In some cases, venue costs can be reduced if a course is hosted at an institution who can provide reduced rates. In addition, the host institution may be able to access reduced local rates for accommodation and transportation. Apart from costs, additional considerations for choosing a location include proximity to airports, ease of transportation and access to course venue, and safety (see Rule 4).

Funding for training is often available through dedicated grants for training or through explicit budget lines in research projects that aim to build bioinformatics capacity. For example, global grant organizations, such as the Wellcome Trust, the Fogarty International Center, and the National Institutes of Health, United States of America [15], can provide funding for training through various funding instruments. Other opportunities may be available from your organization, local funding bodies, or small grants from other regional bodies aimed at training and capacity building. It is prudent to keep a close eye on such opportunities as they may not be broadly advertised and yet could provide modest funds, enabling you to cover important costs for running a course. A collaborative funding model also offers an opportunity to split costs with other funded projects or training organizations. Hosts can negotiate provision of expert trainers in exchange for free or reduced local costs for the venue, transportation, administration, and catering costs. A course fee may also be charged to recover any costs where there are funding constraints or budget limitations, but registration fees should ideally be kept at a minimum to avoid being exclusionary. In cases where course fees become particularly exclusionary, it may also be possible to charge a “no-show” fee by refunding the attendance fee to people who attend.

Methods of payments to secure a place will need to be considered as this may be a hindrance for participants in countries where they cannot process online payments. As a course organizer, you can also identify funding organizations that could sponsor travel and registration bursaries, which applicants can then apply for to increase their chances of attending the course.

### Rule 3: Identify and invite a collaborative team of instructors

Choosing appropriate instructors is a particularly important step in the planning and organization of bioinformatics training in LMICs. You should aim to find and enroll the academic/scientific experts (local or international) that can provide the best possible practical training for the topic of the course. However, the selection of trainers has implications for the budget and training format, which you must consider carefully.

Local trainers are likely to have a deeper understanding of the relevant research areas and available technologies and will also have a greater appreciation of the local customs, language barriers, and challenges associated with successfully delivering the workshop. They may also act as role models for students from underrepresented demographics within science, technology, engineering, and mathematics (STEM), inspire professional development, and establish best practices that enable training sustainability. This is key to avoiding the “parachute” phenomenon common in research and training programs based in LMICs where provision often falls short of building capacity and sustainability [16].

Furthermore, it will be cheaper to invite local trainers as travel and accommodation costs will be much lower compared to those for international trainers.

On the other hand, inviting international instructors with specialist knowledge and expertise may provide a broader learning experience. Collaborating with international instructors also provides a broader scope of learning experience for all, sharing experiences in training activities and styles, networking, and strengthening trainer capacity. International speakers may not, however, be able to deliver training in the local language (a problem where trainees are not confident in learning outside of their first language) and will increase the budget for workshop organization.

In practice, collaborative teams of local and international trainers provide the benefits of having both local and international trainers and provide an important opportunity to foster interactions and build networks [17]. To ensure a greater sustainability of impact, it could be possible to use local teaching assistants to help deliver the workshop alongside international trainers. Training by international instructors could be done in person or using a hybrid setup. International instructors could participate remotely via teleconferencing if the infrastructure for adequate remote training is available at the training site. Such a model could also reduce the cost and logistics of physically bringing international instructors at the expense of the richer interactions between instructors and trainees during face-to-face training. Incorporating a “Train the Trainer” aspect, where local scientists are separately trained to deliver effective training on a particular topic, will further increase this impact.

### Rule 4: Ensure that infrastructure is adequate for training

In LMICs, there may be limited availability of suitable training facilities that provide the necessary infrastructure for delivering a bioinformatics workshop. Ideal locations are equipped with the required technical infrastructure, are accessible for travel, and their use fits within budget costs. The choice of venue will also depend on the context of the training event, which may include training as part of a global training program supported by a global training team, a regional program organized by a consortium training team, or a one-off training event supported through a small grant. Some training may also take place in combination with other events such as conferences. When choosing between potential venues, a combination of site visits and venue checklists (**Table 1**) will be useful to determine the most suitable location. Hotels and convention centers usually have good facilities for hosting workshops; however, for technical workshops, these places are unlikely to provide the required IT infrastructure.

Furthermore, capacity building through networking with collaborators and other scientific personnel is an important consideration for bioinformatics training in LMICs, so choosing an academic or research institute may be the best choice to help foster these interactions. Another advantage for choosing an academic venue is the possibility of recruiting local students to help with organization of the event. They are also extremely enthusiastic to assist with arranging local extracurricular activities important for networking and capacity building.

Once the city and the optimal venue for the workshop are decided, it is advisable to make bookings 6 to 12 months prior to the workshop dates, bearing in mind the various rules of hospitality in making reservations and cancellations.

**Table 1:** Venue checklist: special characteristics that a venue must fulfill to have a fruitful workshop.

Issue	Requirement
Computers	If using computers in a training center, ensure that they have the required operating systems, up-to-date licensed software, and antivirus software is installed. The same is required if participants bring their own laptops.
Technical equipment	Availability of projectors/pointers and any other technical equipment required by trainers. Organizers must contact trainers and make sure they send details of every piece of software and equipment that they will use in the workshop.
Room location	It must be easy to access, with the capacity to accommodate the expected number of participants comfortably and safely
Technical staff	Complete availability of technical staff in case any problems arise during the workshop
Internet	Dedicated broadband internet, extra bandwidth if required and cost, with access to dedicated servers if needed
Accessibility	Facilities for people with disabilities
Local catering	Availability of local catering for coffee breaks and lunch
Alternative rooms	A contingency plan in case there is any major issue with the main training room
Venue booking cost	Usually, there will be costs involved with booking a venue for a workshop and how the costs should be covered.
Accommodation	Organizers must examine the availability of hotels close to the teaching venue; transport can be problematic in major cities so try to avoid long-distance transfers from hotels to teaching venues. Be aware of other events happening around the same time as the training, as lodging gets scarce during big events.

### Rule 5: Design a clear and transparent application process

Due to the lack of training opportunities, courses organized in LMICs are often oversubscribed. Using a “first-come, first-served basis” to selecting attendees is therefore not ideal as the resulting trainee group may not have the skills required to benefit from the training content. A clearly defined target audience, where the actual needs and knowledge gaps are fully known, enables the criteria to select attendees who would benefit most from the training to be clearly stated. A clear and transparent application process is therefore required to ensure that the people who will most benefit from the training can access the course.

Course advertisements should include full details of prerequisite skills and knowledge of attendees along with a clear statement of the selection criteria. Additionally, a clear policy and statement regarding equity, diversity, and inclusion both within the application and selection processes should be available and included. An example of the latter is a gender balance statement, accompanied by gender-inclusive advertisement-wide wording, following United Nations (UN) guidelines [18]. This works to encourage prospective applicants to apply, who may have not otherwise considered submitting an application. Working closely with local organizers throughout this process is a great way to guide decisions that would drive long-term impact for their institution and region.

For an application process to be undertaken, as a minimum requirement, it is necessary to have an application form with a set of questions guided by the course learning outcomes and which allows organizers to understand applicants’ backgrounds, motivation, and justification of need, detailing how the training will benefit them. Additional questions may be included to assess any relevant prerequisite skills and knowledge criteria.

Many institutions or organizations may have in-house resources to develop forms, which should be used where possible. If not, many simple web-based form builder tools exist such as Google Forms, which allow the creation of a simple application form while choosing an appropriate storage location and ability to control access to the data collected. Remember to check for any access restrictions to the application platform you choose and include a data protection statement to clarify use of any data on the application form, emphasizing the privacy of information provided.

To undertake selection of applicants, it is good practice to form a diverse committee of trainers and organizers who will independently review and select successful applications based on the previously agreed upon criteria. Common selection criteria that can be used to improve the short-term and long-term impact of the workshop include demonstrating a need for attending the training, potential for use in analyzing own datasets, and the potential to transfer newly acquired skills to colleagues. The committee should aim to select a diverse group of students, both in terms of background and skill levels, to facilitate peer-to-peer learning, thus enriching the training outcomes for the group as a whole.

Participants should be provided with clear acceptance guidelines and terms and conditions. Applicants who are not selected could be guided toward Findable, Accessible, Interoperable, and Reusable (FAIR) training resources available via online channels such as YouTube and GitHub (Rule 7). In the prevailing virtual training model, it may be possible to have open lecture sessions, enabling a wider audience to access some parts of the course. If the whole course has to be run remotely, e.g., due to travel restrictions, it is important to have local or ongoing support for trainees in a virtual classroom to try to emulate interactions in a classroom as closely as possible.

### Rule 6: Double-check travel requirements and health and safety advice

Generally, it is considered best practice to provide a welcome/travel pack to traveling trainers and participants regardless of the location which they are traveling to. This is particularly important, however, for LMIC regions if there are specific health and safety and/or security risks.

As a course organizer, start by creating a travel checklist that you can then share in the form of a travel pack.

A travel risk assessment should be undertaken and will provide details about health and security risks and how they can be mitigated. You should always seek advice from the government’s official travel offices and country offices, embassies, and travel agents regarding health and safety, vaccinations, entry requirements, and the latest information about events that may impact travel arrangements.

Furthermore, it is important to research travel restrictions for nationalities of traveling trainers and participants to allow sufficient time for preparation of travel documentation. If visas are required, provide guidance on application requirements, timelines, and costs. Sometimes, visas are quick and inexpensive to organize through online portals. For other countries, obtaining a visa may require filling out an application form, obtaining official letters of invitation, biometric data submission in person at your local embassy, a large processing fee, and long waiting periods. Travelers will need to confirm visa categories since bioinformatics training often falls in a gray area between tourism and business. The time and money required to obtain visas should always be factored into the planning and budgeting if the course organizers are fully responsible for providing funds. It is also important to consider whether the accommodation booked for trainers has the required facilities and is located in an area that is safe and within close proximity to the workshop venue to mitigate difficulties with traffic.

Travel to LMICs to deliver training requires safeguarding and close monitoring of one’s health. Several countries have compulsory vaccine certificate requirements such as yellow fever. Additional information can also be found from WHO travel guidelines [19]. Ensure that travelers are also aware of other vaccination and prophylactic recommendations especially for malaria, which is endemic in most LMICs located in tropical regions. A pre- and post-travel health check with a health professional is recommended in case of any underlying conditions or if travelers feel unwell within days of traveling or upon their return. In addition, a basic medical kit containing insect repellent, oral hydration salts, anti-allergy medication, and painkillers is highly recommended. It is advisable to have travel insurance covering health emergencies and to know where to find help and relevant health facilities. Provide details of a local host as a point of contact for emergencies.

This set of information can then be collated into a welcome/travel pack, which should be sent to trainers before their departure date. It should contain all details of their trip, including transport details, accommodation details, local contacts, safety advice, and activities/entertainment popular in the city. It should also contain up-to-date information on prevailing security issues such as civil unrest, crime, or local protests and climatic and environmental issues such as floods and earthquakes. If possible, recommend downloading a travel security mobile app that tracks your location and can alert you on security issues. You may also consider providing a prepaid SIM card to trainers traveling from abroad (including other LMICs) to ensure that they have data connectivity when with you.

Although traveling and meeting people in person are always the best ways to engage, teach, and learn, these are not always possible. Where there are challenges of traveling or serious concerns of health and security risks, consider supporting trainers to deliver their sessions remotely as a great way to mitigate against the risks and environmental impacts associated with traveling [20–22]. For courses that move to more virtual formats, as has been necessary during the current Coronavirus Disease 2019 (COVID-19) pandemic, there are opportunities for blending synchronous and asynchronous learning where attendees work through materials or practicals on their own and then join online sessions with fellow attendees and trainers. This format offers flexibility for those who can’t travel or, for example, have full-time jobs.

### Rule 7: Create course materials tailored to your audience

When creating teaching materials for a workshop in an LMIC, as with any training event, it is important to pitch the content at the appropriate level for the audience to achieve the learning outcomes. Following the needs assessment, it is best practice to combine the selection and/or a pre-course survey outcomes to establish the background knowledge, levels of experience, and learning goals of prospective participants (see Rule 1). This will be helpful in further tailoring course materials, and, where necessary, provide pre-course exercises or other online resources prior to the course.

In order to provide a relevant training experience, instructors should consider presenting data and examples that cover local research interests. For instance, using datasets from local studies will give students a perspective on solving particular problems on their projects. Furthermore, it is paramount for participants to apply the tools and software after the course upon return to their institutions, so the course needs to be tailor-made to the tools, technologies, and resources available in the LMICs. In the absence of appropriate tools and infrastructure, a solution is to make software available as virtual machines or in containers (e.g., Docker or Kubernetes) that can be taken away and run on local machines. If there is a choice of software, use those that are feasible to install and run in LMICs, with a preference for freely available software.

Materials should be made available to participants to take away after the course and should also be findable and accessible to others—following the FAIR principles will increase the breadth and sustainability of the impact of the workshop [23]. In order to do this, course materials can be shared on an institution website, GitHub, or repositories such as Zenodo. Having DOIs for the materials will enable them to be citable; materials should be accompanied by licensing and acknowledgments information. Although not always feasible, translation of transcribed materials into other languages may make them more accessible to researchers in LMICs who are not comfortable with English.

As part of the training materials, instructors should also prepare a training outcomes assessment and feedback form. Such reviews are paramount to improving workshop organization and teaching performance, regardless of whether the workshop will be repeated in the same place, context, or format.

### Rule 8: Be flexible and prepared for problems

When delivering bioinformatics training in an LMIC setting, the classroom infrastructure may be unreliable (although this can also be a non-LMIC issue of course!). Make sure you communicate with local hosts prior to the workshop to fully understand local infrastructure challenges that may impact upon your course delivery [24]. For some, it may be intermittent electricity, and, for others, internet connectivity and data costs. Planning an effective strategy to overcome hurdles when and if faced with them during the training will mitigate issues and ensure that the workshop continues uninterrupted. **Table 2** below summarizes some of the main challenges that can be faced by organizers when delivering training in LMICs, along with some recommended solutions. Be prepared to be flexible in your delivery and associated timings so if you need to jump between solutions you can do so without feeling rushed.

**Table 2:** Potential challenges experienced when delivering training in LMICs and their respective solutions.

Challenges	Potential solutions
Loss of internet	Pre-prepared screenshots, examples, and exercises available offline and not requiring internet, have datasets available on local devices, and use of stand-alone analysis platforms such as eBioKit [25]. Even if it’s not needed in the end, they can prove a powerful learning resource that can be shared with the students and used as a reference in the future.
Loss of electrical power	Availability of a printed workbook, blackboard/whiteboard, and activities not requiring electricity will allow the workshop to continue without disruption during loss of electrical power, and, in any case, offline materials will provide a valuable learning resource for students. Allow time for powering up and logging into computers after a power cut, which can take a long time.
Venue (closed, room change, and technical equipment not working or unavailable)	As in Table 1 for Rule 4, have alternative rooms planned. If possible, arrive early to the venue and test software and hardware running and performance. This will help identify technical issues that can be solved before the students arrive. Plan for no or reduced access to computers. For the former, paper-based exercises or group work; for the latter, ask trainees to share computers or have 1 or 2 spare computers available for students who encounter technical issues. Try designing sessions so the most important lessons have technology-free backups.
Loss of time (late start, unexpected early finish, and other sessions overrun)	Discuss with organizers any break times and how much time is needed for your training, but be flexible with session timings and content—decide which parts of a course are crucial and prioritize as appropriate; have backups and/or asynchronous learning ready. Awareness of cultural issues is also key here, e.g., in providing slightly longer breaks to enable participants to attend prayers or cultural norms in terms of expected break lengths and times for lunch/dinner.

### Rule 9: Consider the bigger picture

In general, training events serve many purposes, mainly in enabling capacity building through teaching/learning new skills, knowledge, and attitudes, but also in expanding collaborative networks and gaining new insights and perspectives on different matters, cultural exchanges, and advocacy. Bioinformatics training in LMICs has many challenges including a shortage of trained bioinformaticians and infrastructural problems such as lack of computers or other technologies [26]. It is important to be aware of these challenges and try to connect LMICs with trainers, resources, and other assistance through networking opportunities, exploiting all the opportunities that training events bring to the table, including networking, advocacy, and cultural exchange.

Courses and workshops often involve organizers, researchers, academics, facilitators, and trainers at different stages of their career and frequently other types of professionals (e.g., industry leaders and representatives), making them the perfect setting for networking and providing the opportunity to exchange ideas and experiences in an environment that is striving to achieve diversity and inclusiveness. These environments encourage collaborative endeavors, ensuring sustainability of impact. Some of the best examples are multinational projects, which have education chapters that are centered on networks of trainers such as “The Carpentries” [27] and “Galaxy” [28].

Networking experiences may also help in building transnational collaborations due to the involvement of people from different countries. According to [29], it is “essential that all scientists learn how to network, because it enhances the visibility of a researcher’s work to others in the field and ultimately boosts a person’s success in the scientific arena.” For this reason, it is important to assign time and activities on your program to encourage networking and give everyone the opportunity to get in touch with each other so they can potentially exchange ideas, form long-lasting professional relationships, and share career advice. Icebreakers, flash talks, poster presentations, and science speed dating are examples of activities that can be integrated into the training event itself, while after-program social events can provide a nonformal environment that fosters networking and cultural exchange. To support these experiences, a healthy teaching and learning environment, and reduce the possibility of cultural conflict, it is also essential to implement and enforce a code of conduct. This code should outline the expected ethical, inclusive, and respectful behaviors during the course by all parties involved (organizers, trainers, students, and helpers). It will also allow the trainers or organizers to take action if this code of conduct is violated.

The training event might also have advocacy purposes, such as supporting a research group or promotion of bioinformatics within non-biotechnological parties. In this context, you may be asked for additional lectures or seminars or attend official and unofficial ceremonies, as well as meeting dignitaries from local authorities, research institutes, or universities.

Cherish the experience and opportunities that the training event will bring in terms of cultural exchange. Do desktop research before traveling to an LMIC to get a sense of the cultural differences, cues, and norms. Engaging in a cultural interchange with your hosts helps you to identify those factors that you need to take in consideration into your syllabus. They may vary from academic events that need to be included in the course program (e.g., opening and closing ceremonies or certificate distribution) to local customs and traditions such as allocating time for prayers.

### Rule 10: Use feedback to ensure sustainable impact

When examining the success of a training course or program, you need to identify your key objectives for evaluation, including determining how well the course was implemented, its perceived value, and its impact. Using course participant feedback and demographic data from applicants to implement improvements in the organization and delivery of future iterations will ensure sustainability of impact, which is critical in LMICs where there is reduced access to bioinformatics training events. This is also important for reporting to funders and institutions who are key stakeholders and expect demonstration of diverse reach, building or strengthening capacity, impact, sustainability, and, ultimately, why the training should continue to receive support. Funding for training activities within LMICs is often quite scarce and highly competitive.

This coupled with various other barriers such as access to training resources, access to skilled trainers, remote locations, and the relatively high costs associated with training activities act as major barriers to delivering training in LMICs. Assessing the impact of training thus becomes incredibly vital especially for securing future funding and support for the training program. To determine outputs and short-term outcomes, data and feedback should be collected before, during, and after the training event [30]. Demographic data (numbers of applicants, career level, sector, and region/country location) collected during the application phase will give an indication of reach and whether you were able to access your intended audience. This data can be used to demonstrate the ongoing need for such training and may help identify additional audiences.

The most commonly applied source of feedback (and easiest to administer) are post-course surveys, which are used to determine the immediate success of a course and feed into its continual improvement. Designed to provide both quantitative and qualitative information, such surveys can be used to learn participant views about the ease and mode of delivery, course/module content, achievement of learning outcomes, support during the course, and ideas about additional learning needs. Course impact on an individual level can be followed up after 6 to 12 months, by surveying to determine how participants are applying their skills in their research or work. Adaptations of evaluation frameworks can be used to assess the effectiveness of training, value creation, learner’s views and experiences, their increase in knowledge, skills and changes in attitudes, and implementation of acquired knowledge or skills in their work [31,32]. It is important to ensure that participants consent to any data collection, and data protection laws are followed.

## Conclusions

**Fig 1:**
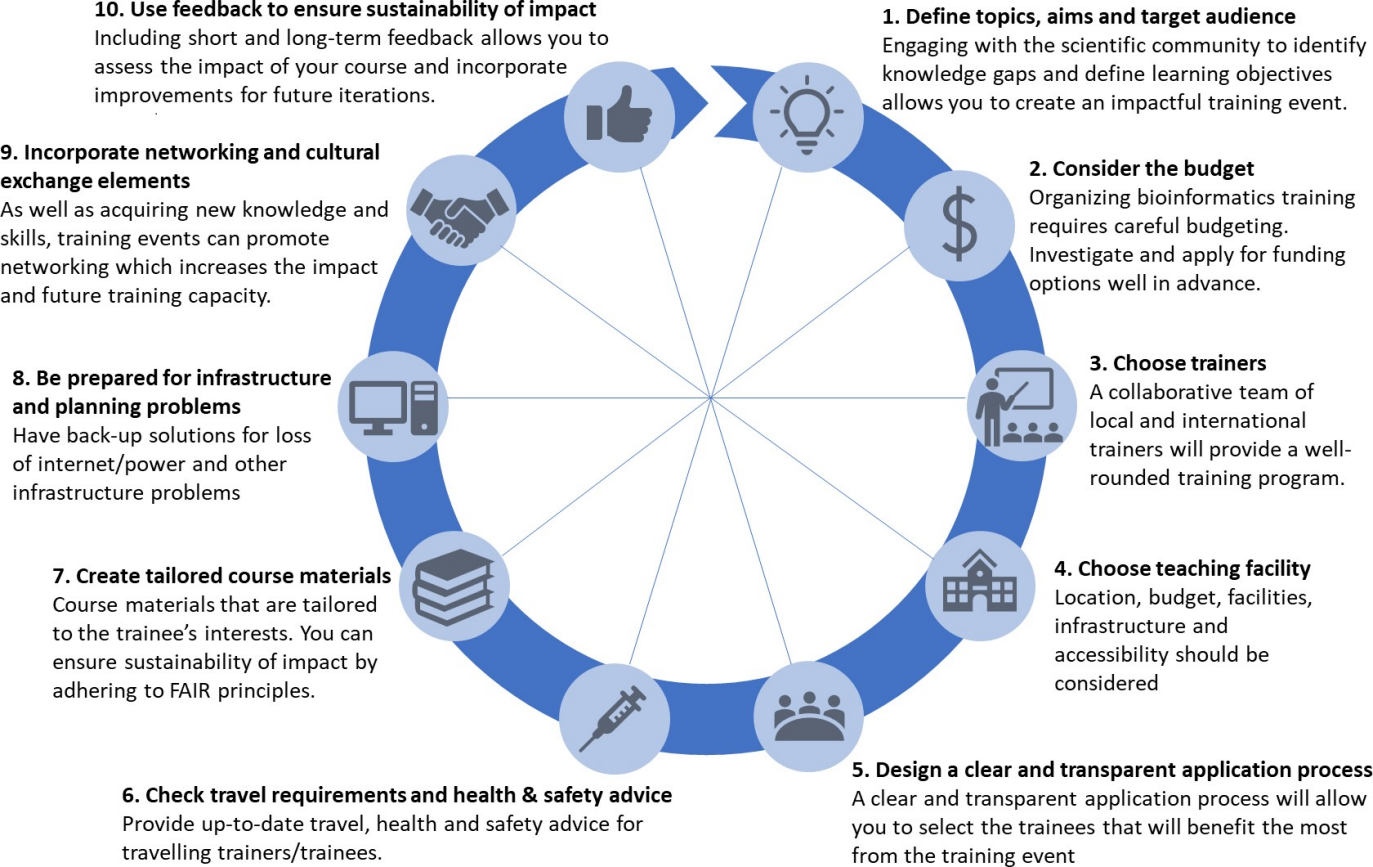
Summary of the 10 simple rules for organizing a bioinformatics training event in an LMIC. FAIR, Findable, Accessible, Interoperable, and Reusable; LMIC, low- and middle-income country.

The demand for bioinformatics and computational biology training in LMICs is increasing concertedly alongside increased research capacity, and this is likely to continue in the years ahead. To sustain this growth in research capacity and ensure that investment in these areas is fruitful, effective training with long-term sustainable impact is of paramount importance. Delivering training in LMICs presents its own set of challenges, but attention to key steps in the organization and delivery can ensure that your training event provides trainees with the knowledge and skills to facilitate their research and build the capacity for continued growth and expansion of the bioinformatics work being carried out in LMICs all around the world. This paper provides guidance on these steps (**Fig 1**) using our experience in providing training in LMICs.
