# Correction: Ten simple rules for organizing a bioinformatics training course in low- and middle-income countries

**DOI:** 10.1371/journal.pcbi.1010197

**Published:** 2022-05-25

**Authors:** Benjamin Moore, Patricia Carvajal-López, Paballo Abel Chauke, Marco Cristancho, Victoria Dominguez Del Angel, Selene L. Fernandez-Valverde, Amel Ghouila, Piraveen Gopalasingam, Fatma Zahra Guerfali, Alice Matimba, Sarah L. Morgan, Guilherme Oliveira, Verena Ras, Alejandro Reyes, Javier De Las Rivas, Nicola Mulder

The funding statement for this article should read as follows:

“The authors who contributed to this manuscript are funded as follows: BM receives salary support from Wellcome Trust grants [WT108749/Z/15/Z, WT108749/Z/15/A], PC, VR, NM, AG’s salaries are funded in whole, or in part, by the NIH Common Fund H3ABioNet grant [U24HG006941], MC, SLFV, AR, PG, PCL’s salaries were partly funded by the UKRI-BBSRC ‘Capacity building for bioinformatics in Latin America’ (CABANA) grant, on behalf of the Global Challenges Research Fund [BB/P027849/1], JDLR is funded by ISCiii AES [ref. PI18/00591] at the CSIC/USAL (Spain) and by CYTED, RIABIO (Red Iberoamericana 521RT0118), AM’s salary is funded by [WT206194/Z/17/Z], GO is funded by the CABANA grant and SM is funded by the EMBL-EBI. For the purpose of open access, the author has applied a CC-BY public copyright license to any Author Accepted Manuscript version arising from this submission. The funders had no role in study design, data collection and analysis, decision to publish, or preparation of the manuscript.”
